# *N*-(3-Chloro­phen­yl)-2-methyl­benzamide

**DOI:** 10.1107/S1600536808010143

**Published:** 2008-04-16

**Authors:** B. Thimme Gowda, Sabine Foro, B. P. Sowmya, Hartmut Fuess

**Affiliations:** a Department of Chemistry, Mangalore University, Mangalagangotri 574 199, Mangalore, India; b Institute of Materials Science, Darmstadt University of Technology, Petersenstrasse 23, D-64287 Darmstadt, Germany

## Abstract

The conformation of the N—H bond in the structure of the title compound, C_14_H_12_ClNO, is *anti* to the *meta*-chloro substituent in the aniline ring, while the C=O bond is *syn* to the *ortho*-methyl substituent in the benzoyl ring. The conformations of the N—H and C=O bonds are *anti* to each other, similar to those observed in 2-methyl-*N*-(3-methyl­phen­yl)benzamide (N3MP2MBA). The –NHC(=O)– group makes a dihedral angle of 55.8 (7)° with the benzoyl ring, while the angle between the benzoyl and aniline rings is 37.5 (1)°; the respective values for N3MP2MBA are 55.2 (7) and 36.2 (1)°. N—H⋯O hydrogen bonds link the mol­ecules into infinite chains running along the *c* axis.

## Related literature

For related literature, see: Gowda *et al.* (2003 ▶, 2008*a* ▶,*b* ▶).
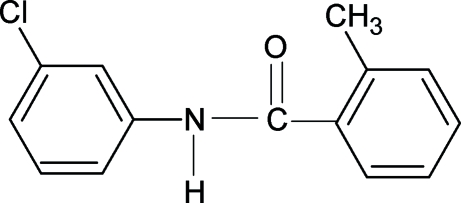


## Experimental

### 

#### Crystal data

C_14_H_12_ClNO
*M* *_r_* = 245.70
Tetragonal, 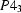

*a* = 8.8237 (8) Å
*c* = 15.977 (2) Å
*V* = 1243.9 (2) Å^3^
*Z* = 4
Cu *K*α radiation
μ = 2.57 mm^−1^
*T* = 299 (2) K
0.60 × 0.10 × 0.07 mm

#### Data collection

Enraf–Nonius CAD-4 diffractometer
Absorption correction: ψ scan (North *et al.*, 1968 ▶) *T*_min_ = 0.308, *T*_max_ = 0.841
4144 measured reflections
2173 independent reflections
1844 reflections with *I* > 2σ(*I*)
*R*_int_ = 0.036
3 standard reflections frequency: 120 min intensity decay: none

#### Refinement

*R*[*F* ^2^ > 2σ(*F* ^2^)] = 0.042
*wR*(*F* ^2^) = 0.120
*S* = 1.07
2173 reflections
159 parameters
1 restraint
H atoms treated by a mixture of independent and constrained refinement
Δρ_max_ = 0.18 e Å^−3^
Δρ_min_ = −0.29 e Å^−3^
Absolute structure: Flack (1983 ▶), 1020 Friedel pairs
Flack parameter: 0.00 (2)

### 

Data collection: *CAD-4-PC* (Enraf–Nonius, 1996 ▶); cell refinement: *CAD-4-PC*; data reduction: *REDU4* (Stoe & Cie, 1987 ▶); program(s) used to solve structure: *SHELXS97* (Sheldrick, 2008 ▶); program(s) used to refine structure: *SHELXL97* (Sheldrick, 2008 ▶); molecular graphics: *PLATON* (Spek, 2003 ▶); software used to prepare material for publication: *SHELXL97*.

## Supplementary Material

Crystal structure: contains datablocks I, global. DOI: 10.1107/S1600536808010143/om2228sup1.cif

Structure factors: contains datablocks I. DOI: 10.1107/S1600536808010143/om2228Isup2.hkl

Additional supplementary materials:  crystallographic information; 3D view; checkCIF report

## Figures and Tables

**Table 1:** Hydrogen-bond geometry (Å, °)

*D*—H⋯*A*	*D*—H	H⋯*A*	*D*⋯*A*	*D*—H⋯*A*
N1—H1N⋯O1^i^	0.83 (4)	2.12 (4)	2.900 (3)	157 (3)

Symmetry code: (i) 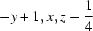.

**Fig. 1.**
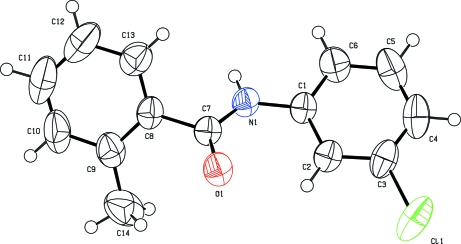
Molecular structure of the title compound, showing the atom labeling. Displacement ellipsoids drawn at the 50% probability level.

**Fig. 2.**
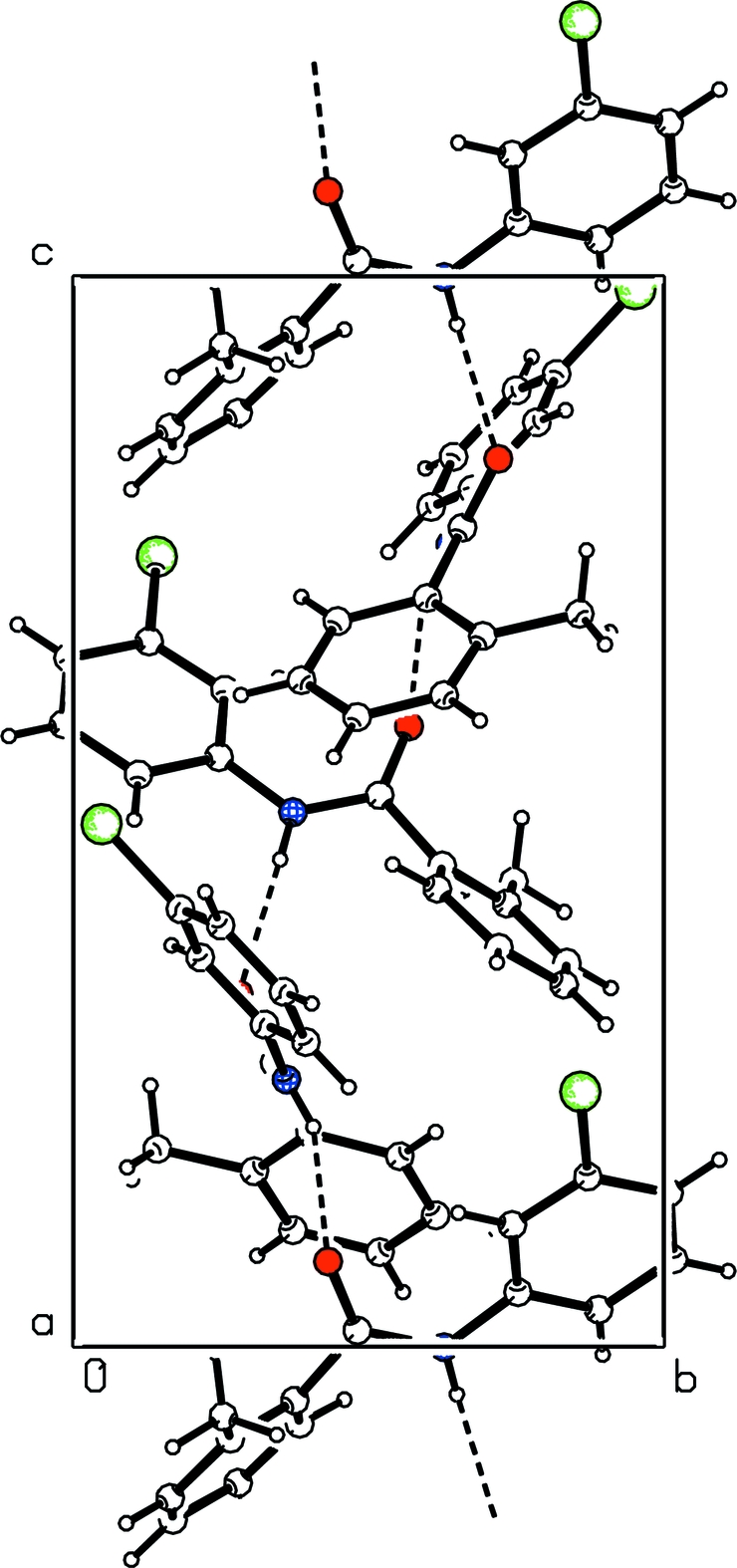
Molecular packing of the title compound as viewed down a and with hydrogen bonding shown as dashed lines.

## supplementary crystallographic information

### Comment

In the present work, the structure of 2-methyl-*N*-(3-chlorophenyl)- benzamide (N3CP2MBA) has been determined as part of substituent effect studies on the solid state structures of benzanilides (Gowda *et al.*, 2003; Gowda *et al.* (2008a, 2008b). The conformation of the N—H bond in N3CP2MBA (Fig. 1) is *anti* to the *meta*-chloro substituent in the aniline ring, while the C=O bond is *syn* to the *ortho*-methyl substituent in the benzoyl ring and the conformations of the N—H and C=O bonds are *anti* to each other, identical to that observed in 2-methyl-*N*-(3-methylphenyl)-benzamide (N3MP2MBA). The bond parameters in N3CP2MBA are similar to those in 2-methyl-*N*-(phenyl)-benzamide (Gowda *et al.*, 2008a), N3MP2MBA (Gowda *et al.*, 2008b) and other benzanilides (Gowda *et al.*, 2003). The amide group, –NHCO– makes a dihedral angle of 55.8 (7)° with the benzoyl ring, while that between benzoyl and aniline rings is 37.5 (1)°, compared to the respective values of 55.2 (7)° and 36.2 (1)° for N3MP2MBA. The packing diagram of N3CP2MBA showing the hydrogen bonds N1—H1N···O1 (Table 1) is given in Fig. 2.

### Experimental

The title compound was prepared according to the literature method (Gowda *et al.*, 2003). The purity of the compound was checked by determining its melting point. It was characterized by recording its infrared and NMR spectra. Single crystals of the title compound were obtained from an ethanolic solution and used for X-ray diffraction studies at room temperature.

### Refinement

The H on N1 was located in difference map and its position freely refined. The other H atoms were positioned with idealized geometry using a riding model with C—H = 0.93–0.96 Å and were refined with isotropic displacement parameters (set to 1.2 times of the *U*_eq_ of the parent atom).

### Crystal data

**Table:**

C_14_H_12_ClNO	*Z* = 4
*M**_r_* = 245.70	*F*_000_ = 512
Tetragonal, *P*4_3_	*D*_x_ = 1.312 Mg m^−^^3^
Hall symbol: P 4cw	Cu *K*α radiation λ = 1.54180 Å
*a* = 8.8237 (8) Å	Cell parameters from 25 reflections
*b* = 8.8237 (8) Å	θ = 5.7–21.9º
*c* = 15.977 (2) Å	µ = 2.57 mm^−^^1^
α = 90º	*T* = 299 (2) K
β = 90º	Rod, colourless
γ = 90º	0.60 × 0.10 × 0.07 mm
*V* = 1243.9 (2) Å^3^	

### Data collection

**Table:**

Enraf–Nonius CAD-4 diffractometer	*R*_int_ = 0.036
Radiation source: fine-focus sealed tube	θ_max_ = 66.9º
Monochromator: graphite	θ_min_ = 5.0º
*T* = 299(2) K	*h* = −10→10
ω/2θ scans	*k* = −10→1
Absorption correction: Psi-scan(North *et al.*, 1968)	*l* = −19→16
*T*_min_ = 0.308, *T*_max_ = 0.841	3 standard reflections
4144 measured reflections	every 120 min
2173 independent reflections	intensity decay: none
1844 reflections with *I* > 2σ(*I*)	

### Refinement

**Table:**

Refinement on *F*^2^	Hydrogen site location: inferred from neighbouring sites
Least-squares matrix: full	H atoms treated by a mixture of independent and constrained refinement
*R*[*F*^2^ > 2σ(*F*^2^)] = 0.042	*w* = 1/[σ^2^(*F*_o_^2^) + (0.0774*P*)^2^ + 0.019*P*] where *P* = (*F*_o_^2^ + 2*F*_c_^2^)/3
*wR*(*F*^2^) = 0.120	(Δ/σ)_max_ = 0.025
*S* = 1.07	Δρ_max_ = 0.19 e Å^−^^3^
2173 reflections	Δρ_min_ = −0.29 e Å^−^^3^
159 parameters	Extinction correction: SHELXL97 (Sheldrick, 2008), Fc^*^=kFc[1+0.001xFc^2^λ^3^/sin(2θ)]^-1/4^
1 restraint	Extinction coefficient: 0.0079 (12)
Primary atom site location: structure-invariant direct methods	Absolute structure: Flack (1983), 1020 Friedel pairs
Secondary atom site location: difference Fourier map	Flack parameter: 0.00 (2)

### Special details

**Table:**

Geometry. All e.s.d.'s (except the e.s.d. in the dihedral angle between two l.s. planes) are estimated using the full covariance matrix. The cell e.s.d.'s are taken into account individually in the estimation of e.s.d.'s in distances, angles and torsion angles; correlations between e.s.d.'s in cell parameters are only used when they are defined by crystal symmetry. An approximate (isotropic) treatment of cell e.s.d.'s is used for estimating e.s.d.'s involving l.s. planes.
Refinement. Refinement of *F*^2^ against ALL reflections. The weighted *R*-factor *wR* and goodness of fit *S* are based on *F*^2^, conventional *R*-factors *R* are based on *F*, with *F* set to zero for negative *F*^2^. The threshold expression of *F*^2^ > σ(*F*^2^) is used only for calculating *R*-factors(gt) *etc*. and is not relevant to the choice of reflections for refinement. *R*-factors based on *F*^2^ are statistically about twice as large as those based on *F*, and *R*- factors based on ALL data will be even larger.

### Fractional atomic coordinates and isotropic or equivalent isotropic displacement parameters (Å^2^)

**Table:**

	*x*	*y*	*z*	*U*_iso_*/*U*_eq_	
C1	0.6762 (3)	0.7532 (3)	0.05087 (15)	0.0463 (6)	
C2	0.7849 (3)	0.7433 (3)	0.11372 (17)	0.0528 (6)	
H2	0.8336	0.6521	0.1249	0.063*	
C3	0.8186 (4)	0.8716 (4)	0.15884 (17)	0.0627 (8)	
C4	0.7505 (5)	1.0087 (4)	0.1436 (2)	0.0763 (10)	
H4	0.7762	1.0940	0.1747	0.092*	
C5	0.6440 (5)	1.0169 (4)	0.0815 (2)	0.0831 (11)	
H5	0.5963	1.1088	0.0705	0.100*	
C6	0.6061 (4)	0.8896 (3)	0.0346 (2)	0.0673 (8)	
H6	0.5337	0.8965	−0.0075	0.081*	
C7	0.6591 (3)	0.4810 (3)	0.01510 (14)	0.0452 (5)	
C8	0.5994 (3)	0.3752 (3)	−0.05047 (17)	0.0516 (6)	
C9	0.6935 (4)	0.2659 (3)	−0.08605 (18)	0.0644 (8)	
C10	0.6279 (6)	0.1643 (4)	−0.1412 (2)	0.0887 (12)	
H10	0.6885	0.0897	−0.1650	0.106*	
C11	0.4808 (7)	0.1690 (4)	−0.1617 (3)	0.0992 (14)	
H11	0.4415	0.0984	−0.1991	0.119*	
C12	0.3864 (5)	0.2793 (5)	−0.1272 (3)	0.0955 (13)	
H12	0.2841	0.2826	−0.1411	0.115*	
C13	0.4472 (4)	0.3834 (4)	−0.0721 (2)	0.0695 (8)	
H13	0.3863	0.4591	−0.0495	0.083*	
C14	0.8575 (5)	0.2539 (5)	−0.0653 (3)	0.0923 (12)	
H14A	0.8693	0.2414	−0.0059	0.111*	
H14B	0.9088	0.3444	−0.0828	0.111*	
H14C	0.9003	0.1680	−0.0936	0.111*	
N1	0.6388 (3)	0.6279 (2)	−0.00053 (14)	0.0488 (5)	
H1N	0.594 (4)	0.650 (3)	−0.045 (2)	0.059*	
O1	0.7197 (2)	0.4315 (2)	0.07953 (11)	0.0598 (5)	
Cl1	0.95205 (13)	0.85850 (13)	0.23813 (6)	0.0996 (4)	

### Atomic displacement parameters (Å^2^)

**Table:**

	*U*^11^	*U*^22^	*U*^33^	*U*^12^	*U*^13^	*U*^23^
C1	0.0581 (14)	0.0483 (13)	0.0324 (12)	−0.0062 (11)	0.0045 (10)	0.0009 (10)
C2	0.0615 (15)	0.0546 (14)	0.0421 (13)	−0.0076 (11)	0.0013 (11)	0.0021 (11)
C3	0.0766 (18)	0.0721 (19)	0.0394 (15)	−0.0252 (15)	0.0044 (13)	−0.0071 (13)
C4	0.115 (3)	0.0605 (18)	0.0538 (19)	−0.0194 (18)	0.0127 (18)	−0.0145 (15)
C5	0.125 (3)	0.0505 (15)	0.074 (2)	0.0089 (18)	0.012 (2)	−0.0043 (16)
C6	0.092 (2)	0.0568 (16)	0.0532 (17)	0.0043 (14)	−0.0034 (16)	0.0023 (13)
C7	0.0528 (13)	0.0500 (13)	0.0327 (13)	−0.0022 (10)	0.0041 (10)	0.0020 (10)
C8	0.0721 (17)	0.0465 (13)	0.0361 (13)	−0.0069 (11)	−0.0036 (11)	0.0030 (10)
C9	0.094 (2)	0.0559 (15)	0.0434 (15)	0.0070 (15)	−0.0037 (14)	−0.0022 (12)
C10	0.149 (4)	0.0572 (18)	0.059 (2)	0.006 (2)	−0.020 (2)	−0.0159 (15)
C11	0.162 (4)	0.064 (2)	0.071 (2)	−0.034 (3)	−0.034 (3)	−0.0049 (18)
C12	0.103 (3)	0.095 (3)	0.089 (3)	−0.039 (2)	−0.039 (2)	0.009 (2)
C13	0.0712 (19)	0.0665 (17)	0.071 (2)	−0.0106 (15)	−0.0094 (15)	0.0044 (15)
C14	0.099 (3)	0.109 (3)	0.069 (2)	0.035 (2)	0.0100 (19)	−0.012 (2)
N1	0.0627 (13)	0.0512 (11)	0.0324 (10)	0.0005 (9)	−0.0063 (10)	0.0014 (9)
O1	0.0893 (13)	0.0559 (10)	0.0342 (10)	0.0054 (9)	−0.0073 (9)	0.0033 (8)
Cl1	0.1114 (7)	0.1195 (8)	0.0678 (6)	−0.0442 (6)	−0.0276 (5)	−0.0081 (5)

### Geometric parameters (Å, °)

**Table:**

C1—C6	1.377 (4)	C8—C13	1.388 (4)
C1—C2	1.391 (4)	C8—C9	1.394 (4)
C1—N1	1.416 (3)	C9—C10	1.384 (5)
C2—C3	1.374 (4)	C9—C14	1.488 (5)
C2—H2	0.9300	C10—C11	1.339 (7)
C3—C4	1.373 (5)	C10—H10	0.9300
C3—Cl1	1.734 (3)	C11—C12	1.394 (7)
C4—C5	1.368 (6)	C11—H11	0.9300
C4—H4	0.9300	C12—C13	1.382 (5)
C5—C6	1.392 (5)	C12—H12	0.9300
C5—H5	0.9300	C13—H13	0.9300
C6—H6	0.9300	C14—H14A	0.9600
C7—O1	1.240 (3)	C14—H14B	0.9600
C7—N1	1.332 (3)	C14—H14C	0.9600
C7—C8	1.499 (3)	N1—H1N	0.83 (4)
			
C6—C1—C2	120.0 (2)	C10—C9—C8	117.3 (3)
C6—C1—N1	117.9 (2)	C10—C9—C14	120.2 (3)
C2—C1—N1	122.0 (2)	C8—C9—C14	122.5 (3)
C3—C2—C1	118.4 (3)	C11—C10—C9	122.8 (4)
C3—C2—H2	120.8	C11—C10—H10	118.6
C1—C2—H2	120.8	C9—C10—H10	118.6
C4—C3—C2	122.6 (3)	C10—C11—C12	120.3 (3)
C4—C3—Cl1	119.1 (2)	C10—C11—H11	119.9
C2—C3—Cl1	118.4 (3)	C12—C11—H11	119.9
C5—C4—C3	118.4 (3)	C13—C12—C11	118.9 (4)
C5—C4—H4	120.8	C13—C12—H12	120.5
C3—C4—H4	120.8	C11—C12—H12	120.5
C4—C5—C6	120.8 (3)	C12—C13—C8	120.0 (4)
C4—C5—H5	119.6	C12—C13—H13	120.0
C6—C5—H5	119.6	C8—C13—H13	120.0
C1—C6—C5	119.7 (3)	C9—C14—H14A	109.5
C1—C6—H6	120.1	C9—C14—H14B	109.5
C5—C6—H6	120.1	H14A—C14—H14B	109.5
O1—C7—N1	123.8 (2)	C9—C14—H14C	109.5
O1—C7—C8	120.8 (2)	H14A—C14—H14C	109.5
N1—C7—C8	115.3 (2)	H14B—C14—H14C	109.5
C13—C8—C9	120.7 (3)	C7—N1—C1	128.3 (2)
C13—C8—C7	118.8 (3)	C7—N1—H1N	117 (2)
C9—C8—C7	120.5 (3)	C1—N1—H1N	115 (2)
			
C6—C1—C2—C3	0.5 (4)	C7—C8—C9—C10	174.9 (3)
N1—C1—C2—C3	177.7 (2)	C13—C8—C9—C14	179.4 (3)
C1—C2—C3—C4	−0.8 (4)	C7—C8—C9—C14	−3.6 (4)
C1—C2—C3—Cl1	179.1 (2)	C8—C9—C10—C11	0.9 (5)
C2—C3—C4—C5	0.7 (5)	C14—C9—C10—C11	179.5 (4)
Cl1—C3—C4—C5	−179.2 (3)	C9—C10—C11—C12	0.0 (6)
C3—C4—C5—C6	−0.4 (5)	C10—C11—C12—C13	0.3 (6)
C2—C1—C6—C5	−0.3 (5)	C11—C12—C13—C8	−1.4 (6)
N1—C1—C6—C5	−177.5 (3)	C9—C8—C13—C12	2.3 (5)
C4—C5—C6—C1	0.2 (6)	C7—C8—C13—C12	−174.7 (3)
O1—C7—C8—C13	121.7 (3)	O1—C7—N1—C1	−1.7 (4)
N1—C7—C8—C13	−56.8 (3)	C8—C7—N1—C1	176.9 (2)
O1—C7—C8—C9	−55.2 (3)	C6—C1—N1—C7	−160.9 (3)
N1—C7—C8—C9	126.2 (3)	C2—C1—N1—C7	21.9 (4)
C13—C8—C9—C10	−2.0 (4)		

### Hydrogen-bond geometry (Å, °)

**Table:**

*D*—H···*A*	*D*—H	H···*A*	*D*···*A*	*D*—H···*A*
N1—H1N···O1^i^	0.83 (4)	2.12 (4)	2.900 (3)	157 (3)

Symmetry codes: (i) −*y*+1, *x*, *z*−1/4.
